# The additive effects of type 2 diabetes mellitus on left ventricular deformation and myocardial perfusion in essential hypertension: a 3.0 T cardiac magnetic resonance study

**DOI:** 10.1186/s12933-020-01138-w

**Published:** 2020-09-30

**Authors:** Xue-Ming Li, Li Jiang, Ying-Kun Guo, Yan Ren, Pei-Lun Han, Li-Qing Peng, Rui Shi, Wei-Feng Yan, Zhi-Gang Yang

**Affiliations:** 1 Department of Radiology, West China Hospital, Sichuan University, 37# Guo Xue Xiang, Chengdu, 610041 Sichuan People’s Republic of China; 2 Department of Radiology, Sichuan Cancer Hospital and Institute, Sichuan Cancer Center, School of Medicine, University of Electronic Science and Technology of China, 55# Lan 4 RenMing Road (South), Chengdu, 610041 Sichuan People’s Republic of China; 3 Department of Radiology, Key Laboratory of Birth Defects and Related Diseases of Women and Children of Ministry of Education, West China Second University Hospital, Sichuan University, 20# South Renmin Road, Chengdu, 610041 Sichuan People’s Republic of China; 4 Department of Endocrinology and Metabolism, West China Hospital, Sichuan University, 37# Guo Xue Xiang, Chengdu, 610041 Sichuan People’s Republic of China

**Keywords:** Hypertension, Type 2 diabetes mellitus, Left ventricular strains, Perfusion, Magnetic resonance imaging

## Abstract

**Background:**

Type 2 diabetes mellitus (T2DM) increases the risks of heart failure and mortality in patients with hypertension, however the underlying mechanism is unclear. This study aims to investigate the impact of coexisting T2DM on left ventricular (LV) deformation and myocardial perfusion in hypertensive individuals.

**Materials and methods:**

Seventy hypertensive patients without T2DM [HTN(T2DM−)], forty patients with T2DM [HTN(T2DM+)] and 37 age- and sex-matched controls underwent cardiac magnetic resonance examination. Left ventricular (LV) myocardial strains, including global radial (GRPS), circumferential (GCPS) and longitudinal peak strain (GLPS), and resting myocardial perfusion indices, including upslope, time to maximum signal intensity (TTM), and max signal intensity (MaxSI), were measured and compared among groups by analysis of covariance after adjusting for age, sex, body mass index (BMI) and heart rate followed by Bonferroni’s post hoc test. Backwards stepwise multivariable linear regression analyses were performed to determine the effects of T2DM on LV strains and myocardial perfusion indices in patients with hypertension.

**Results:**

Both GRPS and GLPS deteriorated significantly from controls, through HTN(T2DM−), to HTN(T2DM+) group; GCPS in HTN(T2DM+) group was lower than those in both HTN(T2DM−) and control groups. Compared with controls, HTN(T2DM−) group showed higher myocardial perfusion, and HTN(T2DM+) group exhibited lower perfusion than HTN(T2DM−) group and controls. Multiple regression analyses considering covariates of systolic blood pressure, age, sex, BMI, heart rate, smoking, indexed LV mass and eGFR demonstrated that T2DM was independently associated with LV strains (GRPS: *p *= 0.002, model *R*^2^= 0.383; GCPS: *p *< 0.001, model *R*^2^= 0.472; and GLPS: *p *= 0.002, model *R*^2^= 0.424, respectively) and perfusion indices (upslope: *p *< 0.001, model *R*^2^= 0.293; TTM: *p *< 0.001, model *R*^2^= 0.299; and MaxSI: *p *< 0.001, model *R*^2^= 0.268, respectively) in hypertension. When both T2DM and perfusion indices were included in the regression analyses, both T2DM and TTM were independently associated with GRPS (*p *= 0.044 and 0.017, model *R*^2^= 0.390) and GCPS (*p *= 0.002 and 0.001, model *R*^2^= 0.424), and T2DM but not perfusion indices was independently associated with GLPS (*p *= 0.002, model *R*^2^= 0.424).

**Conclusion:**

In patients with hypertension, T2DM had an additive deleterious effect on subclinical LV systolic dysfunction and myocardial perfusion, and impaired myocardial perfusion by coexisting T2DM was associated with deteriorated LV systolic dysfunction.

## Introduction

Given their common risk factors, essential hypertension and type 2 diabetes mellitus (T2DM) frequently coexist. Approximately 70% of patients with T2DM have hypertension, and the development of T2DM is almost 2.5 times more likely in patients with precedent hypertension [1, 2]. It is well known that both hypertension and T2DM are well-established risk factors for cardiovascular disease morbidity and mortality regardless of the presence of each factor [3]. In addition, T2DM in hypertensive patients further increases the risks of heart failure and all-cause and cardiovascular mortality [4, 5]. Once established, heart failure is associated with worse clinical outcomes. Therefore, early detection of subclinical left ventricular (LV) myocardial dysfunction allows for earlier intervention, which may potentially prevent heart failure and improve patient outcomes.

Cardiac magnetic resonance (CMR) has less shortcomings of echocardiography, such as acoustic window limitations, low spatial resolution and high operator dependency, and can evaluate cardiac anatomy and function as well as myocardial perfusion in a single examination [6]. The commonly used global volumetric measurement of left ventricular ejection fraction (LVEF) cannot provide a detailed assessment of cardiac mechanics, and it has been demonstrated to be an insensitive and late marker of contractile impairment [7]. Recently, CMR feature tracking using routinely acquired cine images has become a more sensitive technique for evaluating global and regional myocardial deformation as an indicator of subclinical myocardial dysfunction [8]. In addition, CMR-derived first-pass myocardial perfusion has been increasingly used to noninvasively evaluate myocardial microcirculation function with high reproducibility [9, 10].

Although limited echocardiographic studies have observed more severe subclinical systolic dysfunction in patients with coexisting hypertension and T2DM than in those with T2DM alone [11–14], none have evaluated the impact of T2DM on LV deformation and myocardial microcirculation function in patients with hypertension. Accordingly, the aim of this study was to investigate the impact of coexisting T2DM on subclinical LV systolic dysfunction and myocardial microcirculation function using CMR in patients with hypertension.

## Materials and methods

### Study population

**Fig. 1:**
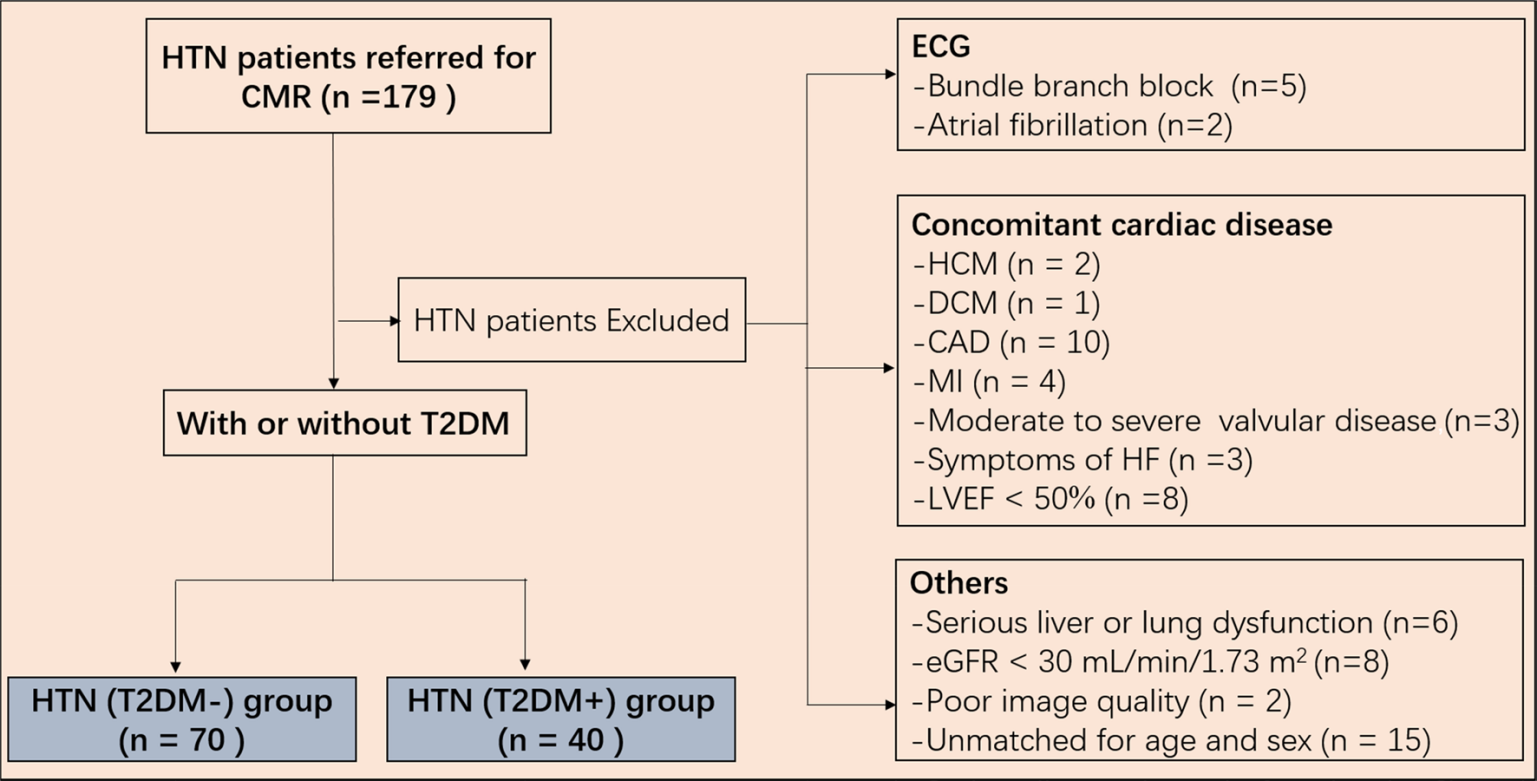
Flow diagram of the study patients. CMR, cardiac magnetic resonance; HTN, hypertension; T2DM, type 2 diabetes mellitus; HCM, hypertrophic cardiomyopathy; DCM, dilated cardiomyopathy; CAD, coronary artery disease; MI, myocardial infarction; HF, heart failure; LVEF, left ventricular ejection fraction; eGFR, estimated glomerular filtration rate

From January 2016 to January 2020, 179 Adult Chinese Han race essential hypertensive patients with or without T2DM [HTN(T2DM+) and HTN(T2DM−), respectively] who underwent CMR at our institution were consecutively included. Hypertension was defined as a clinical systolic blood pressure (SBP) ≥ 140 mmHg and/or a diastolic blood pressure (DBP) ≥ 90 mmHg or a history of antihypertensive medications. The diagnosis of T2DM was based on the current American Diabetes Association guideline recommendations [15]. The exclusion criteria (Fig. 1) included patients with symptoms of heart failure (n = 3), LVEF < 50% (n = 8), known coronary artery disease (n = 10), myocardial infarction (n = 4), moderate to severe valvular disease (n = 3), cardiomyopathy (n = 3), bundle branch block (n = 5), atrial fibrillation (n = 2), serious liver and lung dysfunction (n = 6), estimated glomerular filtration rate (eGFR) < 30 mL/min/1.73 m^2^ (n = 8) and poor image quality (n = 2). In addition, patients unmatched for age and sex (n = 15) were also excluded. Finally, 110 patients including 70 (35 men and 35 women; mean age, 55.0 ± 14.1 years) and 40 (20 men and 20 women; mean age, 55.7 ± 9.8 years) age- and sex-matched patients with HTN (T2DM−) and HTN (T2DM+), were eligible for this study. Another 37 healthy individuals (18 men and 19 women; mean age, 54.2 ± 10.5 years) matched for age and sex were selected from our healthy volunteer database to serve as the control group, and they underwent the same CMR examination.

This study was approved by the Biomedical Research Ethics Committee of our hospital and conducted in accordance with the Declaration of Helsinki (2013 EDITION).

### CMR protocol

All the CMR examinations were performed using a 3.0 T whole-body scanner (Trio Tim; Siemens Medical Solutions, Erlangen, Germany) in the supine position. Data acquisition was performed with a standard ECG-triggering device that monitored each subject’s dynamic ECG changes during the end-inspiratory breath hold period. A balanced steady-state free precession (bSSFP) sequence (repetition time [TR]: 39.34 ms, echo time [TE]: 1.22 ms, flip angle: 40°, slice thickness: 8 mm, field of view [FOV]: 250 × 300 mm, and matrix size: 208 × 139) was used to acquire 8 - 12 continuous cine images from the base to the apex in the short-axis view, as well as vertical LV two- and four-chamber cine images in the long-axis view. For perfusion imaging, a dose of 0.2 mL/kg gadobenate dimeglumine (MultiHance 0.5 mmol/mL; Bracco, Milan, Italy) was injected into the right antecubital vein with a power injector (Stellant, MEDRAD, Indianola, PA, USA) at a flow rate of 2.5–3.0 mL/s, followed by 20 mL of saline. Rest first-pass perfusion images were acquired in three standard short-axis slices (basal, middle, and apical) and in one four-chamber view slice by inversion recovery prepared echo-planar imaging sequence (TR/TE: 163.0/1.12 ms, flip angle: 10°, slice thickness: 8 mm, FOV: 360 mm × 270 mm, and matrix size: 256 × 192). To exclude myocardial infarction, late gadolinium enhancement (LGE) images were acquired by segmented-turbo-FLASH–phase-sensitive inversion recovery (PSIR) sequences (TR/TE: 750 ms/1.18 ms; flip angle: 40°, slice thickness: 8 mm, FOV: 400 × 270 mm, and matrix size: 256 × 148) 10–15 min after contrast administration.

### CMR data analysis

CMR images were evaluated using offline commercially available software (cvi42, v. 5.10.2; Circle Cardiovascular Imaging, Calgary, Canada) by two radiologists with more than 3 years of CMR experience, who were blinded to the clinical data.

The endocardial and epicardial contours of the LV myocardium on the short-axis cine images were manually traced at the end-diastolic and end-systolic phases in the cvi42 short-3D module. Then, LV mass at end-diastole, LV end-diastolic volume (LVEDV), LV end-systolic volume (LVESV), LVEF, stroke volume and cardiac index were computed automatically. The trabeculae and papillary muscles were excluded from the LV mass and included in the LV cavity. LV mass, LVEDV and LVESV indexed for body surface area (BSA) (LVMI, LVEDVI and LVESVI, respectively) were calculated using the Mosteller formula [16]. In addition, LV remodeling index, calculated as LVM/LVEDV, was included for analysis.

The LV global radial (GRPS), circumferential (GCPS) and longitudinal peak strain (GLPS) were obtained by manually delineating the endocardium and epicardium of the cine images at the end-diastole from the short-axis and long-axis two- and four-chamber slice views in the tissue tracking module. Strain was depicted as relative lengthening, shortening and thickening of the myocardium from end diastole (reference phase).

**Fig. 2:**
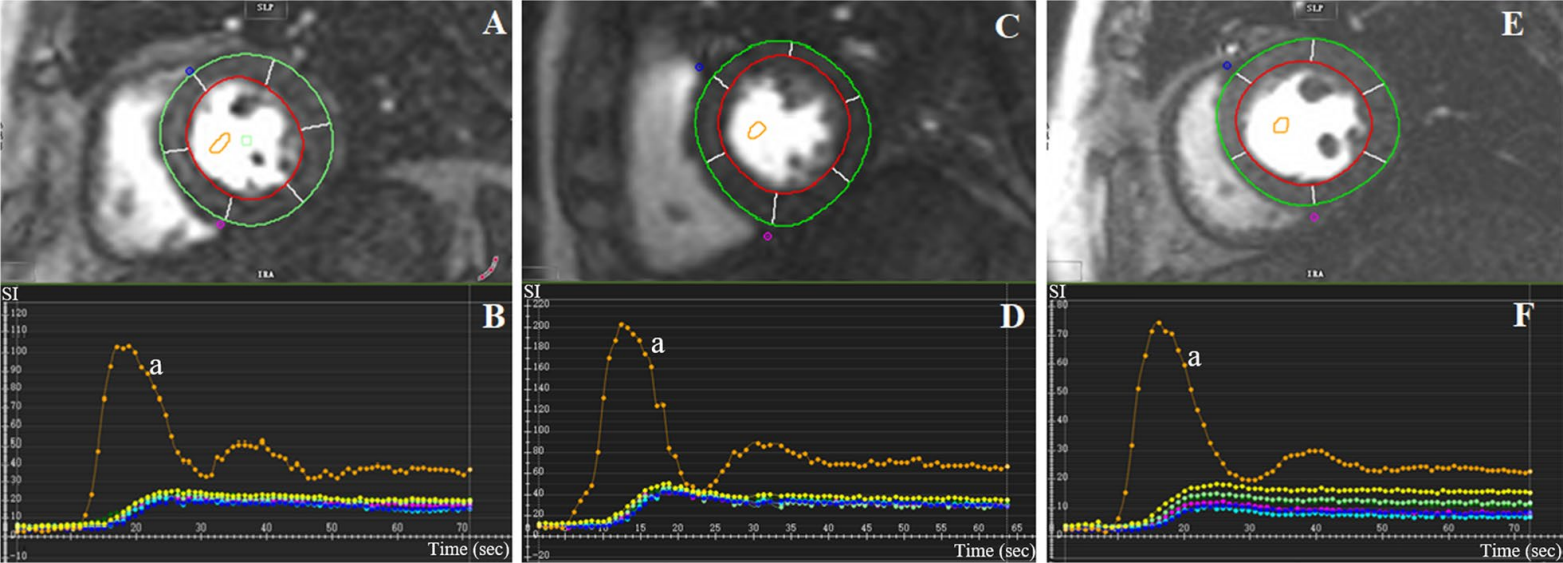
Representative first-pass myocardial perfusion MR images and signal intensity-time curves obtained from left mid-ventricular slice in normal controls (**A**, **B**), patients with HTN (T2DM−) (**C**, **D**) and HTN (T2DM+) (**E**, **F**). The orange curve (a) represents blood-pooled time-signal intensity curve and other colour curves represent time-signal intensity curves in each myocardial segment. The x-axis represents time and y-axis represents signal intensity

For the evaluation of first-pass myocardial perfusion (Fig. 2), the endocardium and epicardium and a region of interest drawn in the LV chamber were manually determined in the first-pass perfusion images (basal, middle and apical). Then, signal intensity-time curves were generated for the blood pool and each myocardial segment based on the 16-segment heart model. Consequently, semiquantitative segmental perfusion indices including the upslope, time to maximum signal intensity (TTM), and max signal intensity (MaxSI) were acquired automatically, and the global first-pass myocardial perfusion indices for each subject were calculated by averaging the regional values of the 16 myocardial segments. In addition, the presence of LGE was visually evaluated by the two radiologists with consensus.

### Reproducibility of LV myocardial strain and perfusion

Intra- and inter-observer variabilities for the LV global myocardial strain and perfusion indices were analyzed in 30 random cases including 20 HTN patients and 10 controls. To determine the intra-observer variability, one observer (XM. L) evaluated the same subjects on two separate measurements 1 month apart. For the inter-observer variability evaluation, a second investigator (L. J) who was blinded to the first observer’s results and clinical data reanalyzed the measurements.

### Statistical analysis

Categorical variables are presented as frequencies (percentages) and were compared using Chi square tests. Continuous variables were evaluated for normality distribution by the Shapiro–Wilk test and are expressed as the mean ± standard deviation (SD). One-way analysis of variance (one-way ANOVA) was used to compare the baseline characteristics among the normal and HTN groups. Comparisons of the CMR-derived parameters between different groups were evaluated by analysis of covariance (ANCOVA) after adjusting for age, sex, body mass index (BMI) and heart rate followed by Bonferroni’s post hoc test. Pearson’s correlation coefficient was used to determine the correlation between LV myocardial strain and first-pass myocardial perfusion indices in HTN. Backwards stepwise multivariable linear regression analyses were performed to determine the predictors for LV strains and myocardial perfusion indices in the whole population and patients with HTN. Inter- and intra-observer agreements were determined by the evaluation of intraclass correlation coefficients (ICCs). All analyses were performed in SPSS version 21 (IBM, Armonk, New York, USA), and a two-tailed *p* < 0.05 was considered significant.

## Results

### Baseline characteristics

The main clinical characteristics of the study participants are summarized in Table 1. Age, BSA, heart rate, plasma triglycerides, total cholesterol, high-density lipoprotein cholesterol, low-density lipoprotein cholesterol and eGFR were not significantly different between the observed groups (all *p* > 0.05), except for a higher BMI in both the HTN (T2DM−) and HTN (T2DM+) groups than in the control group (all *p* < 0.01). As expected, fasting blood glucose was significantly higher in the HTN (T2DM+) group than in the HTN (T2DM−) group and control group (all *p* < 0.001). In addition, both SBP and DBP were significantly higher in the hypertensive groups than in the control group (all *p* < 0.001).

**Table 1:** Baseline characteristics of the study cohort

	Controls	HTN (T2DM−)	HTN (T2DM+)
	n = 37	n = 70	n = 40
Demographics			
Age (years)	54.2 ± 10.5	55.0 ± 14.1	55.7 ± 9.8
Male; n (%)	19 (51.4)	35 (50.0)	20 (50.0)
BMI (kg/m^2)^	23.00 ± 2.74	24.76 ± 3.01*	25.04 ± 2.20*
BSA (kg/m^2^)	1.70 ± 0.18	1.71 ± 0.18	1.74 ± 0.16
Smoking, n (%)	0	27 (38.6)	12 (30.0)
Laboratory data			
Fasting blood glucose (mmol/L)	5.61 ± 1.67	5.30 ± 1.01	7.98 ± 2.67^§^
Plasma triglycerides (mmol/L)	1.34 ± 0.53	1.76 ± 1.28	1.92 ± 1.64
Total cholesterol (mmol/L)	4.71 ± 1.04	4.39 ± 1.04	4.22 ± 0.80
HDL (mmol/L)	1.44 ± 0.40	1.40 ± 1.12	1.21 ± 0.28
LDL (mmol/L)	2.82 ± 0.97	2.52 ± 0.81	2.38 ± 0.67
eGFR (mL/min/1.73 m^2^)	98.4 ± 14.5	93.6 ± 18.8	90.2 ± 18.8
Hemodynamic variables			
Heart rate (beats/min)	70.1 ± 9.9	72.9 ± 16.7	71.5 ± 10.1
SBP (mmHg)	114.2 ± 13.2	141.1 ± 19.5*	138.7 ± 14.5*
DBP (mmHg)	71.6 ± 8.5	87.7 ± 16.5*	84.6 ± 9.6*
HTN treatment			
ACEI/ARB, n (%)	0	28 (40.0)	14 (35.0)
Beta-blocker, n (%)	0	27 (38.6)	16 (40.0)
Calcium channel blocker, n (%)	0	39 (55.7)	19 (47.5)
Diuretics, n (%)	0	10 (14.3)	6 (15.0)
Diabetes treatment			
Oral, n (%)	0	0	33 (82.5)
Insulin, n (%)	0	0	7 (17.5)

The values are the mean ± SD, Numbers in the brackets are percentages

HTN, hypertension; T2DM, type 2 diabetes mellitus; BMI, body mass index; BSA, body surface area; HDL, high-density lipoprotein cholesterol; LDL, low-density lipoprotein cholesterol; eGFR, estimated glomerular filtration rate; ACEI, angiotensin converting enzyme inhibitor; ARB, angiotensin II receptor blocker

* *p* < 0.005 versus controls

^§^*p* < 0.001 versus controls and HTN (T2DM−) group

### Comparison of CMR findings among groups

The CMR findings for the observed groups are shown in Table 2. The LVMI (*p* < 0.001 and = 0.005, respectively) and LV remodeling index (*p* < 0.001 and = 0.040, respectively) in both the HTN (T2DM−) and HTN (T2DM+) groups were significantly higher than those in the control group, whereas the LVEDVI, LVESVI, LVEF, stroke volume and cardiac index were not significantly different among the groups (all *p* > 0.05).

**Table 2:** Comparisons of CMR findings between controls, HTN (T2DM−) group and HTN (T2DM+) group after adjustment for age, sex, BMI and heart rate

	Controls	HTN (T2DM−)	HTN (T2DM+)	*P* value
	n = 37	n = 70	n = 40	
LV geometry and function				
LVMI (g/m^2^)	44.55 ± 8.06	58.07 ± 16.02*	55.22 ± 13.22*	< 0.001
LVEDVI (mL/m^2^)	75.92 ± 12.42	78.26 ± 17.00	76.95 ± 13.51	0.223
LVESVI (mL/m^2^)	28.51 ± 7.62	28.65 ± 10.71	29.39 ± 9.02	0.437
LVEF (%)	62.85 ± 6.20	63.84 ± 7.04	63.00 ± 7.66	0.408
Stroke volume (mL)	78.09 ± 12.04	83.99 ± 21.10	80.18 ± 14.66	0.082
Cardiac index (L/min/m^2^)	3.27 ± 0.60	3.70 ± 0.86	3.37 ± 0.72	0.068
LV remodeling index (g/mL)	0.60 ± 0.12	0.75 ± 0.17*	0.73 ± 0.18*	0.001
Myocardial strain				
GRPS (%)	38.98 ± 8.14	36.57 ± 10.81*	31.77 ± 10.91*^§^	< 0.001
GCPS (%)	− 21.44 ± 2.74	− 21.41 ± 3.10	− 19.88 ± 3.59*^§^	0.004
GLPS (%)	− 14.94 ± 2.42	− 13.14 ± 2.66*	− 11.75 ± 3.69*^§^	< 0.001
Myocardial perfusion				
Upslope	2.73 ± 1.17	3.30 ± 1.29*	1.83 ± 0.74*^§^	< 0.001
TTM (s)	26.90 ± 10.15	23.30 ± 8.78	31.65 ± 12.57*^§^	0.002
MaxSI	23.03 ± 8.03	27.10 ± 9.64*	18.05 ± 6.78^§^	< 0.001

HTN, hypertension; T2DM, type 2 diabetes mellitus;LV, left ventricular; M, mass; EDV, end diastolic volume; ESV, end systolic volume; I, indexed to BSA; EF, ejection fraction; GRPS, global radial peak strain; GCPS, global circumferential peak strain; GLPS, global longitudinal peak strain; TTM, time to maximum signal intensity; MaxSI, max signal intensity

* *p* < 0.05 versus controls

^§^*p* < 0.05 versus HTN (T2DM−) group

The LV GRPS and GLPS (all *p *< 0.05 and 0.027, respectively) declined significantly from controls, through HTN (T2DM−) group, to HTN(T2DM+) group. Compared with the control group, the LV GCPS was decreased in the HTN(T2DM+) group (*p* = 0.005) but preserved in the HTN (T2DM−) group.

Compared with the control group, higher myocardial perfusion was observed in the HTN (T2DM−) group as supported by increased upslope (*p *= 0.023) and MaxSI (*p *= 0.005), and lower myocardial perfusion was demonstrated in the HTN (T2DM+) group as supported by decreased upslope (*p *= 0.036) and increased TTM (*p *= 0.038). In addition, the HTN (T2DM+) group exhibited worse myocardial perfusion than the HTN (T2DM−) group, which was supported by a reduced upslope and MaxSI (all *p *< 0.001) and increased TTM (all *p *= 0.001) values.

### Association among LV strain, myocardial perfusion and clinical variables in the whole population

Multivariable linear regression analyses demonstrated that considering the covariates of SBP, age, sex, BMI, BSA, heart rate, SV and LVM, both hypertension and T2DM were independently associated with LV GLPS (*β* = 1.469 and 1.569, *p* = 0.006 and 0.003, model *R*^2^ = 0.467), and T2DM but not hypertension was independently associated with LV GCPS and GRPS (*β* = 1.553, *p* = 0.002, model *R*^2^ = 0.442 and *β* = − 5.493, *p* = 0.001, model *R*^2^ = 0.425, respectively). In addition, both hypertension and T2DM were independently associated with upslope (*β* = 0.608 and − 1.437, *p* = 0.009 and < 0.001, model *R*^2^ = 0.293) and MaxSI (*β* = 4.346 and − 9.223, *p* = 0.014 and < 0.001, model *R*^2^ = 0.232), and T2DM but not hypertension was independently associated with TTM (*β *= 5.878, *p* = 0.001, model *R*^2^ = 0.371).

### Association between myocardial strain and first-pass myocardial perfusion in hypertension

LV GRPS, GCPS and GLPS were significantly associated with upslope and TTM, whereas they were not associated with MaxSI (Table 3).

**Table 3:** Correlation of LV myocardial strain with first-pass perfusion indices in all patients with hypertension

	Upslope		TTM (s)		Max SI	
	*r*	*p* value	*r*	*p* value	*r*	*p* value
GRPS (%)	0.292	0.003	− 0.355	<0.001	0.092	0.362
GCPS (%)	− 0.226	0.024	0.390	<0.001	− 0.067	0.508
GLPS (%)	− 0.299	0.002	0.279	0.005	− 0.144	0.152

Multivariable linear regression analyses (Table 4) revealed that considering the covariates of SBP, age, sex, BMI, heart rate, smoking, LVMI and eGFR, T2DM was independently associated with LV strains (GRPS: *β* = − 6.178, *p* = 0.002, model *R*^2^ = 0.383; GCPS: *β* = 2.314, *p* < 0.001, model *R*^2^ = 0.472; and GLPS: *β* = 1.685, *p* = 0.002, model *R*^2^ = 0.424, respectively) and first-pass myocardial perfusion indices (upslope: *β* = − 1.448, *p* < 0.001, model *R*^2^= 0.293; TTM: *β *= 8.188, *p* < 0.001, model *R*^2^= 0.299; and MaxSI: *β* = − 9.325, *p* < 0.001, model *R*^*2*^ = 0.268, respectively). Furthermore, when both T2DM and all the perfusion indices were included in the regression analyses, both T2DM and TTM were independently associated with LV GRPS (*β* = − 4.233, *p* = 0.044; *β* = − 0.221, *p* = 0.017; model *R*^2^= 0.390) and LV GCPS (*β* = 1.868, *p* = 0.002; *β* = 0.054, *p* = 0.001; model *R*^2^= 0.495), and T2DM but not perfusion indices was independently associated with LV GLPS (*β *= 1.685, *p* = 0.002, model *R*^2^= 0.424).

**Table 4:** Multivariable association of diabetes with first-pass myocardial perfusion indices or LV strains in all patients with hypertension adjusted for SBP, age, sex, BMI, heart rate, smoking, LVMI and eGFR

Model 1	TTM		Upslope		MaxSI	
	Coefficient (95% CI)	*R*^*2*^	Coefficient (95% CI)	*R*^*2*^	Coefficient (95% CI)	*R*^*2*^
Diabetes	8.188 (4.063 to 12.312) *	0.299	− 1.448 (− 1.952 to − 0.944) *	0.293	− 9.325 (− 13.117 to − 5.533) *	0.268

	GRPS		GCPS		GLPS	
	Coefficient (95% CI)	*R*^2^	Coefficient (95% CI)	*R*^2^	Coefficient (95% CI)	*R*^2^
Model 2						
Diabetes	− 6.178 (− 10.059 to − 2.297)*	0.383	2.314 (1.231 to 3.398)*	0.472	1.685 (0.621 to 2.750)*	0.424
Model 3						
Diabetes	− 4.233 (− 8.343 to − 0.123)*	0.390	1.868 (0.718 to 3.018)*	0.495	1.685 (0.621 to 2.750*	0.424
TTM	− 0.221 (− 0.401 to − 0.041)*		0.054 (0.001 to 0.107)*		–	
Upslope	–		–		–	
MaxSI	–		–		–	

Abbreviation of SBP, BMI and eGFR are shown in Table 1; and LVMI, LVEDVI, GRPS, GCPS, GLPS, TTM and MaxSI in Table 2

Model 1: Association between diabetes and perfusion indices

Model 2: Association between diabetes and LV strains

Model 3: Association of LV strains with diabetes and perfusion indices

* p < 0.05; values are unstandardized estimate coefficients (B) and 95% confident interval (CI)

### Intra-observer and inter-observer variability

As demonstrated in Table 5, there were excellent intra- and inter-observer agreements in the measurement of LV global myocardial peak strain (ICC = 0.934–0.972 and 0.938–0.957, respectively) and first-pass myocardial perfusion (ICC = 0.912–0.931 and 0.891–0.956, respectively).

**Table 5:** Intra-and inter-observer variability of LV strains and perfusion indices

	Intra-observer		Inter-observer	
	ICC	95% CI	ICC	95% CI
GRPS	0.934	0.891–0.954	0.945	0.857–0.989
GCPS	0.972	0.915–0.991	0.957	0.945–0.994
GLPS	0.938	0.878–0.988	0.938	0.852–0.978
Upslope	0.931	0.920–0.986	0.915	0.895–0.983
TTM (s)	0.912	0.905–0.975	0.891	0.840–0.932
MaxSI	0.920	0.869–0.945	0.956	0.869–0.978

Abbreviation of GRPS, GCPS, GLPS, TTM and MaxSI are shown in Table 2

ICC, intraclass correlation coefficient; CI, confidence interval

## Discussion

The main findings of this study are as follows: (1) hypertension impairs LV GLPS, and coexisting T2DM further deteriorates subclinical LV systolic dysfunction; (2) myocardial perfusion is increased in HTN (T2DM−) but decreased in HTN (T2DM+) patients; and (3) impaired myocardial perfusion by T2DM is associated with aggravated LV systolic dysfunction in patients with hypertension. These changes observed in our study indicate the deleterious effect of T2DM on myocardial systolic function and myocardial microcirculation function in patients with hypertension, which may contribute to the increased cardiovascular risk.

### T2DM aggravate LV dysfunction in hypertension

Similar to most progressive myocardial diseases, subendocardial fibers are more vulnerable to being affected; thus, longitudinal contractile function representing as GLPS may be impaired earlier and more severely [8], as shown in previous studies with hypertension [17–19] and T2DM [13, 20–22], as well as in our patients. In hypertension, there is insulin resistance and activation of the sympathetic nervous system and renin–angiotensin–aldosterone system, which may result in diffuse myocardial fibrosis [12]. Diffuse myocardial fibrosis and hemodynamic overload-associated LV hypertrophy are more vulnerable to the involvement of subendocardial fibers. In addition, studies in hypertension have found that GCPS was significantly reduced in patients with hypertrophy [17, 23]. Recent studies showed the negative effects of acute hyperglycemia on systolic LV global longitudinal strain and multilayer longitudinal and circumferential strain in asymptomatic T2DM and unfavorable subclinical reductions in global and average circumferential strain in obese adolescents with dysglycemia, which may indicate the importance of intensive blood glucose control [24, 25]. Moreover, epicardial adipose tissue may be associated with LV structural and functional abnormalities and exercise intolerance in T2DM patients with asymptomatic heart failure [26], and serum levels of omentin-1 and Zinc-α2-glycoprotein have been recently defined as the most important predictors for LV hypertrophy and LV diastolic dysfunction in T2DM patients [27]. Our study demonstrated that T2DM aggravated LV systolic dysfunction as represented by more severe impairment of GLPS and GRPS and the occurrence of impaired GCPS in the HTN (T2DM+) group, even though similar LVEF and LV geometries were observed between the hypertensive subgroups. The reason for this may be the superimposed factors that cause contractile dysfunction in T2DM, such as impairments in excitation–contraction coupling, metabolic derangements, remodeling of the extracellular matrix and abnormalities in microvasculature [28]. Nevertheless, it may be difficult to determine the exact individual contribution, since some of them are usually found together.

### The combined effect of T2DM and hypertension on microvascular dysfunction

In both hypertension and T2DM, there are structural and functional abnormalities in coronary microvasculature, including hypertrophic remodeling of small arteries and arterioles, microvascular rarefaction, and functional increases in vasoconstriction due to endothelial dysfunction [2, 28, 29]. However, resting myocardial perfusion reflecting autoregulated blood flow correlates with myocardial oxygen consumption and is mainly determined by LV wall stress, myocardial contractility and heart rate [1]. Increased myocardial perfusion was observed in our HTN (T2DM−) patients, which was consistent with previous studies [30–32]. Kjaer et al. [31] demonstrated a 25% higher baseline myocardial perfusion by positron emission tomography, and Kozakova et al. [30] revealed higher resting coronary flow in the left anterior descending artery by transesophageal Doppler echocardiography. The increased resting myocardial perfusion in hypertension may reflect an adaptive mechanism, adapting to the increased oxygen demand for the heart to work under increased afterload [29, 32]. In T2DM, increased resting myocardial perfusion has been exhibited in some previous studies [33–35], while others have reported decreased resting perfusion [20, 36]. This discrepancy may be due to different study populations, investigation modalities, and relatively modest sample sizes. Increased plasma insulin, a known vasodilator, by insulin resistance in T2DM may explain the increased myocardial perfusion at rest [34]. Additionally, glucose and lactate oxidation is inhibited, and fatty acid oxidation is increased, which may lead to more oxygen consumption, subsequently resulting in higher resting perfusion [34, 35].

To our knowledge, reports about the combined effect of hypertension and T2DM on myocardial microcirculation are still missing. We found that patients with HTN (T2DM+) showed worsened myocardial perfusion than those with HTN (T2DM−) and control group. A possible explanation for this may be that although hypertension and T2DM increase resting myocardial perfusion to meet increased oxygen consumption, their coexistence may amplify the abovementioned abnormalities of microvasculature that have reduced myocardial perfusion at rest.

### Association between impaired myocardial perfusion and LV dysfunction

In our study, there was a significant correlation between subclinical LV systolic dysfunction and impaired myocardial perfusion, and impaired myocardial perfusion by T2DM was associated with the deterioration of subclinical LV systolic dysfunction in patients with hypertension. Our results were consistent with the study by Jiang et al. [37], which may suggest that myocardial microcirculation might be associated with myocardial systolic function, giving further support to the importance of efficient energy production in normal myocardial contraction [28]. The aggravated abnormalities of the microvasculature discussed above may compromise nutrient and oxygen delivery and energy production, impair myocardial contractility and eventually lead to LV systolic dysfunction. Therefore, pharmacologic treatment aimed at increasing myocardial microvascular function might be an effective method for improving myocardial contractility and preventing heart failure, which needs further study.

### Limitation

Several limitations in this study merit comment. First, this was a cross-sectional single-center study with a relatively small sample size, and further multicenter studies with a larger population should be performed to validate our findings. Second, The PROCEED study revealed that duration of T2DM and systolic blood pressure are determinants of severity of coronary stenosis in asymptomatic diabetes [38]. Although not all our patients underwent coronary computed tomography angiography or invasive coronary angiography, coronary artery disease was considered to be unlikely according to the evaluation of patients by clinical history, laboratory results, echocardiography and electrocardiography which was subsequently supported by the CMR examinations. Third, stress tests were not performed in our participants; thus, myocardial systolic function and perfusion reserve could not be evaluated, and subclinical coronary artery disease was excluded [39]. Even if subclinical coronary artery disease could not be excluded, which may subsequently cause impaired myocardial perfusion and myocardial dysfunction, our results could still reflect the impact of T2DM and hypertension after adjustment for confounders including age. Finally, follow-up observation was not conducted to examine whether changes in myocardial microcirculation over time affect myocardial structure and function, and further longitudinal studies are required to investigate the potential prognostic value of impaired myocardial perfusion and deformation in patients with coexisting hypertension and T2DM.

## Conclusion

In patients with hypertension, T2DM had an additive deleterious effect on subclinical LV systolic dysfunction and myocardial perfusion. In addition, impaired myocardial perfusion by coexisting T2DM was associated with deteriorated LV systolic dysfunction, which may contribute to increased adverse outcomes.

## Data Availability

The datasets used and analyzed during the current study are available from the corresponding author on reasonable request.

## Abbreviations

HTN: Hypertension
T2DM: Type 2 diabetes mellitus
LV: Left ventricular
CMR: Cardiac magnetic resonance
EDV: End-diastolic volume
ESV: End-systolic volume
LVEF: Left ventricular ejection fraction
LVMI: LV mass index
GRPS: Global radial peak strain
GCPS: Global circumferential peak strain
GLPS: Global longitudinal peak strain
TTM: Time to maximum signal intensity
MaxSI: Max signal intensity
